# Detection of HIV type 1 mutations on the pol region in untreated patients in Northern Vietnam: determination of drug resistance and subtypes

**DOI:** 10.1186/1758-2652-13-S4-P127

**Published:** 2010-11-08

**Authors:** S Ranger-Rogez, M Al Jawhari, B Nguyen, N Pham Hong, T Tran Quoc, J Pascual, J Doll, M Harzic, G Viretto, P Weinbreck

**Affiliations:** 1 CHU Dupuytren, Faculté Pharmacie, Virology, Limoges, France; 2 Saint-Paul Hospital, Hanoi, Vietnam; 3 Dong Da Hospital, Hanoi, Vietnam; 4 Centre Hospitalier, Médecine, Versailles, France; 5 Centre Hospitalier, Virologie, Versailles, France; 6 ESTHER, Paris, France; 7 Centre Hospitalier Universitaire, Maladies Infectieuses Tropicales, Limoges, France

## Purpose of the study

The first antiretroviral therapy was introduced in Vietnam in 1990 and included two nucleoside reverse transcriptase inhibitors (NRTIs). More recently, non-nucleoside reverse transcriptase inhibitors (NNRTIs) and protease inhibitors (PIs) were available, particularly through different programmes. In this context, it is interesting to survey the HIV drug resistance and also to determine the main subtypes circulating in this country.

## Methods

Plasmas from 56 seropositive patients were collected before treatment. All patients originated from the area of Hanoi, North Vietnam. After RNA extraction using Nuclisens® easyMag® (Biomérieux), the sequencing of the pol region was conducted according to the ANRS recommendations. The DNA was sequenced on both strands with ABI Prism 3130xl and analyzed with SeqScape software to determine the differences compared to HXB2 strain: determination of resistance was done using the ANRS rules. ClustalX and Treeview software allowed determining the subtype of each strain.

## Summary of results

Among the 56 patients, two revealed HIV resistance to the main NRTIs and NNRTIs (mutations Y181C and Q151M for one patient, and G190A, Q151M, Y115FY and K65R for the other). No resistance was observed for the PIs, except for tipranavir; almost all strains exhibited the association of M36I, H69K and L89M mutations. Concerning the integrase inhibitors, two patients (different from those exhibiting resistance for NRTIs and NNRTIs) revealed the mutation E157Q conferring a resistance to raltegravir. All the strains but one belonged to the subtype CRF15_01B; the last one exhibited a CRF08_BC subtype. Numerous polymorphic mutations were observed among these strains.

## Conclusions

In this study, 2/56 (3, 6%) patients exhibited a resistance to the main NRTIs and NNRTIs before administration of treatment. The same percentage of patients had strains resistant to the raltegravir. The CRF15_01B is the subtype the most prevalent in the population studied.

